# Proton Beam Therapy for Testicular Seminoma: Clinical Outcomes and Toxicity From the Proton Collaborative Group Prospective Registry

**DOI:** 10.7759/cureus.110756

**Published:** 2026-06-12

**Authors:** Lifei Zhu, Keyur J Mehta, Charles B Simone, Arpit M Chhabra, Kristin Hsieh, J. Isabelle Choi, Madhur K Garg, Justin Tang, Arpi D Thukral, Henry K Tsai, Irini Yacoub

**Affiliations:** 1 Radiation Oncology, Montefiore Medical Center/Albert Einstein College of Medicine, Bronx, USA; 2 Radiation Oncology, New York Proton Center, New York, USA; 3 Radiation Oncology, Northwestern Medicine Cancer Center and Proton Center, Warrenville, USA; 4 Radiation Oncology, ProCure Proton Therapy Center, Somerset, USA

**Keywords:** proton beam therapy, proton radiation, radiation-induced toxicity, radiation therapy, testicular seminoma

## Abstract

Introduction: Testicular seminoma predominantly affects young men, and while photon-based radiotherapy achieves excellent disease control, concerns about late effects, including secondary malignancies, have driven interest in proton beam therapy (PBT) as a way to improve dose conformality and spare normal tissue. Prospective clinical data for PBT in seminoma, however, remain scarce.

Methods: We reviewed 19 consecutive patients with histologically confirmed testicular germ cell tumors treated with PBT between 2010 and 2024 from the Proton Collaborative Group (PCG) multi-institutional prospective registry. Primary endpoints included overall survival (OS), progression-free survival (PFS), local control, and toxicity graded per Common Terminology Criteria for Adverse Events (CTCAE) version 5.0.

Results: All 19 patients (19/19, 100%) completed their prescribed radiation course without interruption (median dose 32.8 GyRBE; median 17 fractions). Among 10 patients (10/19, 53%) with evaluable follow-up (median 23.9 months), two-year OS and PFS were both 100% in non-metastatic patients (n=9), with 100% local control (9/9) across the evaluable cohort. One death (1/2 metastatic patients) occurred in a patient with metastatic disease at presentation. Acute toxicities were predominantly grade 1-2 (fatigue, nausea, radiation dermatitis), and all resolved without treatment interruption. No treatment-related grade ≥3 adverse events (0/19) or secondary malignancies were observed. Late toxicities were observed in two of 19 patients (2/19, 11%), with only one late event (grade 1 urinary urgency) attributable to radiation in the non-metastatic cohort. No secondary malignancies were observed during 28.8 person-years of follow-up, comparing favorably to predicted excess rates of two to eight per 100 person-years reported for photon-based approaches.

Conclusions: These results support PBT as a feasible and well-tolerated option for testicular seminoma. A longer follow-up is needed to assess secondary malignancy risk reduction.

## Introduction

Testicular cancer represents the most common solid malignancy in men between 20 and 34 years of age, with seminoma accounting for approximately half of all testicular germ cell tumors [1]. Although its incidence has steadily increased over recent decades, outcomes remain highly favorable, with cure rates exceeding 95% when treated using appropriate combinations of surgery, radiation therapy (RT), and systemic treatment [2]. As a result, attention has increasingly shifted from short-term disease control to the long-term consequences of treatment in this predominantly young and otherwise healthy population.

Following radical inguinal orchiectomy, management options for testicular seminoma include active surveillance, systemic chemotherapy, and RT, with the choice depending on disease stage and risk factors. For stage I disease, active surveillance has become a widely adopted approach, while adjuvant RT remains a standard option for stage IIA-IIB seminoma and select stage I patients, with traditional approaches using photon-based techniques achieving excellent disease control rates [3]. However, the treatment volumes required for para-aortic and pelvic nodal irradiation inevitably expose surrounding normal tissues to low and intermediate radiation doses. With extended follow-up, this exposure has been associated with an increased risk of late adverse effects, including secondary malignancies, cardiovascular disease [4], and other chronic treatment-related complications that may emerge decades after therapy [5,6].

Proton beam therapy (PBT) has been proposed as a means of reducing these long-term risks by limiting radiation dose to non-target tissues. Owing to the finite range of protons and the absence of exit dose beyond the target, proton therapy allows for highly conformal dose distributions that can substantially reduce integral dose compared with photon-based techniques. Prior dosimetric analyses have suggested reductions in normal tissue doses on the order of 50-70% [7]. These properties are especially appealing in seminoma, where radiation fields frequently encompass radiosensitive organs, including the kidneys, bowel, bone marrow, and, in some cases, the heart and lungs.

Despite these advantages, clinical experience with proton therapy for testicular seminoma remains limited. Early reports have shown technical feasibility and encouraging short-term disease control [8,9]; however, published data largely consist of small, single-institution or multi-institution retrospective series with limited follow-up [10,11], and none have reported prospective registry-based outcomes with systematic acute and late toxicity characterization. As a result, real-world data on toxicity profiles, long-term safety, and durability of disease control remain sparse, and the place of proton therapy relative to modern photon techniques is still not well established.

Here we report our multi-institutional experience with PBT for testicular seminoma, focusing on clinical outcomes, treatment-related toxicities, and short-term safety. We hypothesized that proton therapy would achieve disease control comparable to photon-based series while offering a more favorable toxicity profile.

## Materials and methods

Study design and patient population

Consecutive patients were identified from the Proton Collaborative Group (PCG) prospective registry between October 2010 and October 2024. Eligible patients were those with histologically confirmed germ cell tumors of the testis who received proton radiotherapy with curative or palliative intent following radical orchiectomy. Mixed histology was permitted per registry protocol. For the purposes of survival analysis, patients were stratified by treatment intent (curative vs. palliative) and metastatic status at presentation. Curative-intent non-metastatic patients were analyzed separately, with the smaller palliative-intent and metastatic cohorts reported descriptively.

Clinical staging data were extracted from the registry’s baseline diagnosis form, which captures both formal American Joint Committee on Cancer (AJCC) Tumor, Node, Metastasis (TNM) staging and a separate metastatic status indicator. Where a formal group stage was not entered, it was derived from available TNM components when sufficient data were present.

Treatment planning and delivery

All patients underwent computed tomography (CT) simulation in the supine position with appropriate immobilization. Target volumes were delineated according to institutional guidelines and contemporary consensus recommendations for seminoma. Clinical target volumes typically included para-aortic lymph nodes from T11 to L5, with inclusion of ipsilateral iliac nodes depending on disease stage and extent.

Proton therapy was delivered using pencil beam scanning (PBS) or uniform scanning techniques. Treatment planning was performed using commercial treatment planning systems with standard range and setup uncertainties. Quality assurance was performed according to institutional protocols. Prescribed dose and fractionation were summarized as the cumulative course dose (sum across all treatment phases) and total number of fractions.

Data collection and endpoints

Patient demographics, tumor characteristics, treatment details, and outcomes were extracted from electronic medical records. Primary endpoints included overall survival (OS), progression-free survival (PFS), and local control. Secondary endpoints included acute and late toxicities, graded according to Common Terminology Criteria for Adverse Events (CTCAE) version 5.0, secondary malignancy rates, and functional outcomes. For the purposes of this analysis, acute toxicity was defined as adverse events with onset during the radiation course or within 90 days of RT completion; late toxicity was defined as onset more than 90 days after RT completion.

Staging data completeness varied across enrollment periods and contributing sites, reflecting the multi-institutional nature of the PCG registry and evolution of data collection forms over the 14-year study period.

Statistical analysis

Descriptive statistics were used to characterize patient demographics and treatment variables. Survival outcomes were estimated using the Kaplan-Meier method. Toxicities were summarized using frequency tables and percentages. All analyses were performed using Python 3.11 (Python Software Foundation, Beaverton, OR, USA) with the pandas, scipy, and lifelines libraries. Figures were generated using matplotlib and seaborn.

## Results

Patient characteristics

Nineteen male patients with a median age of 39 years (interquartile range (IQR) 35-49) were treated during the study period. Patient demographics and disease characteristics are summarized in Table 1. The cohort was predominantly seminoma (18/19, 95%); one palliative-intent patient had non-seminomatous germ cell tumor histology. The majority of patients were White (14/19, 74%) and had primary testicular disease (17/19, 89%), whereas two patients (2/19, 11%) had metastatic disease at presentation. Among patients with documented staging information, clinical stages included IA, IB, IIA, IIB, and IIIC. Most patients (13/19, 68%) had Eastern Cooperative Oncology Group (ECOG) performance status 0, with two (2/19, 11%) having ECOG 1 and four (4/19, 21%) having no recorded ECOG status before proton therapy. Eighteen patients (18/19, 95%) underwent prior radical orchiectomy.

**Table 1:** Baseline patient and disease characteristics IQR, interquartile range; ECOG, Eastern Cooperative Oncology Group; NSGCT, non-seminomatous germ cell tumor; RT, radiation therapy

Characteristic	Value
Age at diagnosis, median	39 years (IQR 35-49; range 22-72)
Race	White 14 (74%)
	Black 1 (5%)
	Not reported 4 (21%)
Ethnicity	Not Hispanic 13 (68%)
	Hispanic 1 (5%)
	Not reported 5 (26%)
Histology	Seminoma 18 (95%)
	NSGCT 1 (5%)
Clinical stage	IA 1 (5%)
	IB 1 (5%)
	IIA 5 (26%)
	IIB 4 (21%)
	IIIC 1 (5%)
	IV 1 (5%)
	Not reported 6 (32%)
Metastatic status (M stage)	M0: 17 (89%)
	M1: 2 (11%)
ECOG performance status	0: 13 (68%)
	1: 2 (11%)
	Not reported 4 (21%)
Prior orchiectomy	Yes 18 (95%)
	No 1 (5%)
Prior chemotherapy	Yes 3 (16%)
	No 15 (79%)
	Not reported 1 (5%)
Prior RT	Yes 1 (5%)
	No 17 (89%)
	Not reported 1 (5%)
Other prior therapy	Yes 2 (11%)
	No 16 (84%)
	Not reported 1 (5%)

Treatment characteristics

Treatment details are presented in Table 2. All patients (19/19, 100%) completed their prescribed radiation course without interruption. Seventeen patients (17/19, 89%) received curative-intent proton therapy, and two (2/19, 11%) received palliative-intent treatment. Two patients presented with metastatic disease, including one who received curative-intent irradiation and one who received palliative-intent treatment. PBS was the most commonly used technique (10/19, 53%), followed by uniform scanning (3/19, 16%); delivery modality was not recorded for five patients (5/19, 26%), and was not applicable for one patient (1/19, 5%) whose plan phases utilized non-proton techniques.

**Table 2:** Treatment characteristics ᵃ"Not applicable" indicates that the treatment plan did not utilize proton therapy for that phase. "Not recorded" indicates that the delivery modality was not entered in the registry. ᵇTIP, paclitaxel + ifosfamide + cisplatin; BEP, bleomycin + etoposide + platinum; FOLFOX, folinic acid + fluorouracil + oxaliplatin. GyRBE, Gray relative biological effectiveness; IQR, interquartile range; BED₁₀, biological effective dose; EQD2, equivalent dose in 2-Gy fractions; PBS, pencil beam scanning; 3D-CRT, three-dimensional conformal radiation therapy

Parameter	Value
Total dose, median	32.8 GyRBE (IQR 29.95-36.13 Gy; range 25.4-80 Gy)
Number of fractions, median	17 (IQR 15-18; range 10-28)
Dose per fraction (Gy), median	2.0 (IQR 1.73-2.01; range 1.49-8.00)
Plan site categories	Pelvic±para-aortic 3 (16%)
	Para-aortic nodes 10 (53%)
	Testis 1 (5%)
	Metastatic visceral 1 (5%)
	Other 4 (21%)
Boost/secondary plan site categories	Para-aortic boost 4 (31%)
	Pelvic/iliac boost 4 (31%)
	Nodal boost 4 (31%)
	Other boost 1 (8%)
Proton delivery modality	PBS 10 (53%)
	Uniform scanning 3 (16%)
	Not applicable 1 (5%)ᵃ
	Not recorded 5 (26%)ᵃ
Prior chemotherapy	Agents:
	Patient 1: cisplatin + etoposide
	Patient 2: cisplatin + etoposide, TIP, FOLFOXᵇ
	Patient 3: BEP, TIP, etoposide + carboplatinᵇ
BED₁₀ (Gy, α/β=10), mean	41.3±10.8; range 29.2-72.0
EQD2 (Gy), mean	33.0±6.7; range 24.3-49.7
Combined modality	1 patient received a combination of protons and 3D-CRT

**Figure 1:**
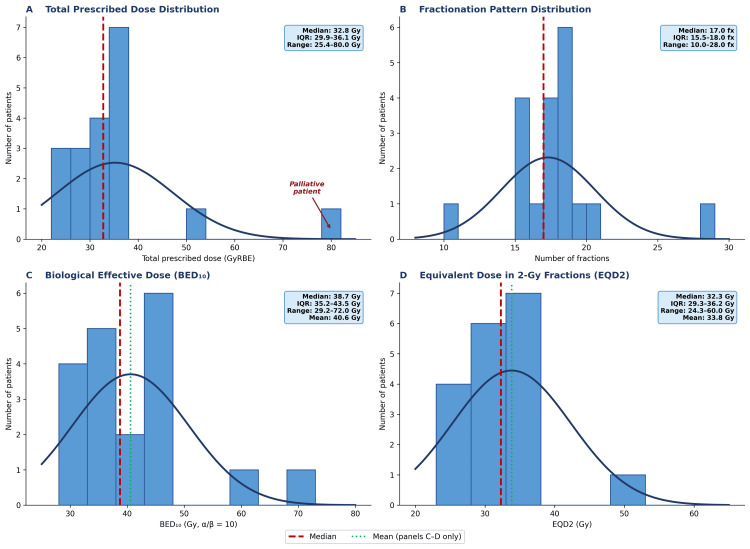
Treatment dose and fractionation distributions (A) Histogram of total prescribed dose (GyRBE) across all 19 patients; the dashed line indicates the median (32.8 GyRBE). The palliative patient receiving 80 GyRBE is annotated separately. (B) Histogram of fractionation patterns; the majority of patients received 15-18 fractions (median: 17). (C) Biologically effective dose (BED₁₀, Gy, α/β=10) distribution with a superimposed normal distribution curve; values clustered between 35.2 and 43.5 Gy₁₀. (D) Equivalent dose in 2-Gy fractions (EQD2) distribution; most patients had EQD2 values of 25-40 GyRBE, consistent with conventional fractionation schemes for seminoma. Red dashed lines = median; green dotted lines = mean. IQR, interquartile range; BED₁₀, biological effective dose; EQD2, equivalent dose in 2-Gy fractions

The median total dose was 32.8 GyRBE (IQR, 30.0-36.1) delivered in a median of 17 fractions (IQR, 15-18) (Figure 1A, 1B). These values reflect cumulative course prescriptions across phases and include both curative-intent and palliative-intent treatments. Median dose per fraction was 2.00 GyRBE (IQR, 1.73-2.01). The biological effective dose (BED₁₀) was centered around 40-45 GyRBE, with the equivalent dose in 2-Gy fractions (EQD2) showing most doses between 30 and 40 GyRBE (Figure 1C, 1D). Fifty-eight percent (11/19) of treatment plans utilized standard fractionation schemes commonly employed for seminoma (20-26 Gy in 10-17 fractions). Para-aortic nodal irradiation (with or without pelvis coverage) was the most common treatment site (13/19, 68%). Prior chemotherapy was administered in three patients (3/19, 16%).

Clinical outcomes

Clinical outcomes are summarized in Table 3. Of 19 patients treated, 10 (10/19, 53%) had evaluable follow-up data for survival analysis. With a median follow-up of 23.9 months (IQR, 13.9-45.2), one patient died during follow-up at 28.3 months after developing distant metastases. The cause of death was not attributed in the available records, although this patient had distant progressive disease at the time of his death.

**Table 3:** Survival outcomes All survival estimates in Section A are exact (100% observed). 95% CIs were calculated using the Clopper-Pearson exact binomial method; for n=9 with zero events, the lower bound=(0.025)^(1/9)=66.4%. Section B patients are reported individually; no aggregate statistics are presented for this subgroup. OS, overall survival; PFS, progression-free survival; CI, confidence interval; IQR, interquartile range; NSGCT, non-seminomatous germ cell tumor

Parameter	Events/total	1-year	2-year	3-year	Median	95% CI (2-year)	Notes
Curative-intent, non-metastatic patients (n=17 treated; n=9 with evaluable follow-up)							
OS	0 deaths/9	100%	100%	100%	Not reached	66.4-100%	No deaths observed
PFS	0 events/9	100%	100%	100%	Not reached	66.4-100%	No local, regional, or distant progression
Local control	9/9 (100%)	-	-	-	-	66.4-100%	No local or regional recurrences
Freedom from distant progression	9/9 (100%)	-	-	-	-	66.4-100%	No distant metastases observed
Median follow-up	-	-	-	-	23.9 months	4.2-98.7 mo	8 patients followed >12 months
Metastatic patients (n=2 treated; n=1 with follow-up) (descriptive)							
Patient 001-000222	1 death/1	-	-	-	28.3 months	-	Metastatic; curative intent; died of distant progression
Patient 018-000942	No follow-up	-	-	-	-	-	Palliative intent; NSGCT; metastatic; excluded from survival analysis

**Figure 2:**
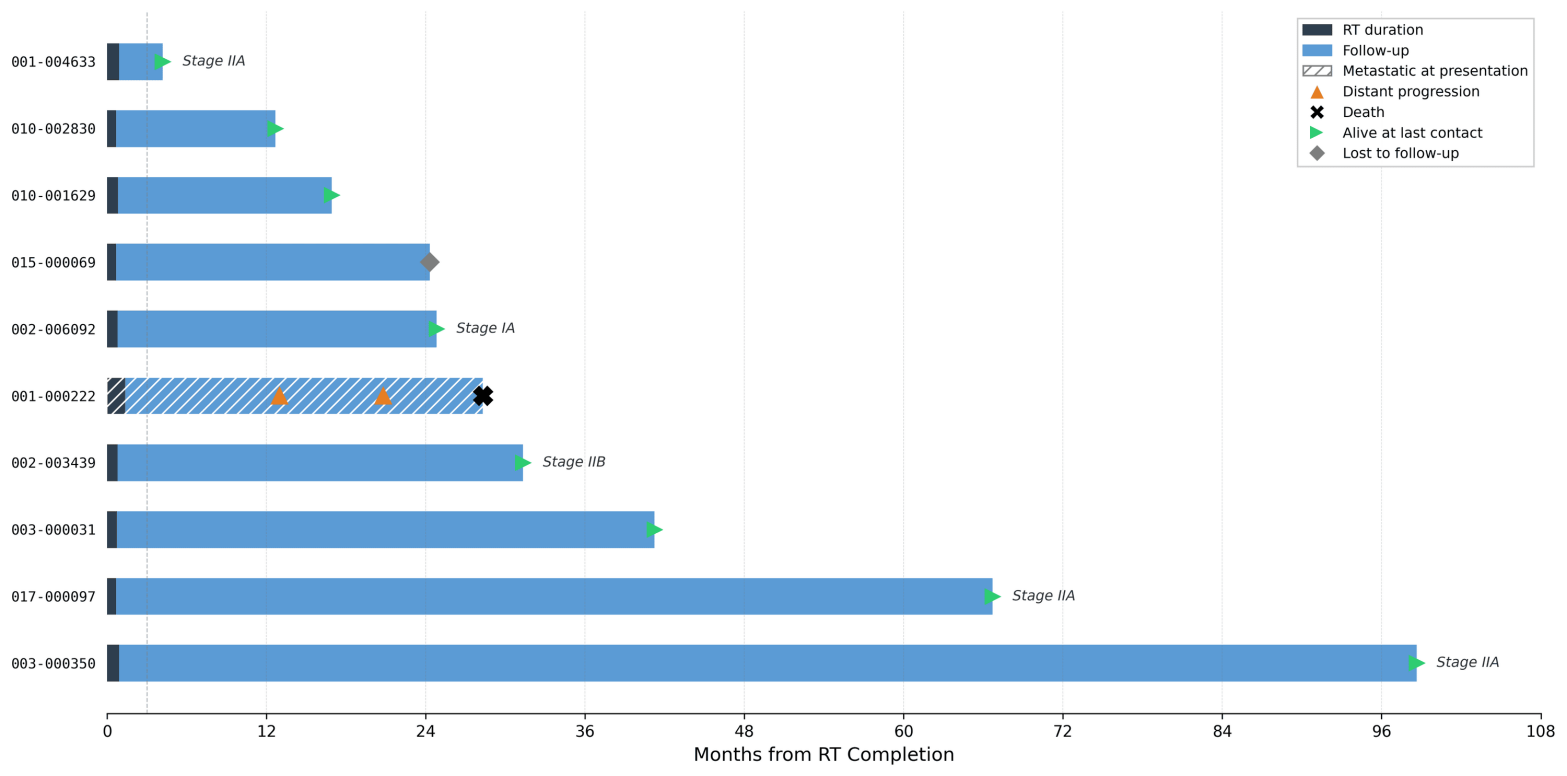
Individual patient follow-up after PBT for testicular seminoma Each bar represents one patient, ordered by follow-up duration. The dark segment at the left of each bar indicates the RT duration. Hatched pattern = metastatic disease at presentation. Symbols: ► alive at last contact; ✕ death; ▲ distant progression; ◆ lost to follow-up. The dashed vertical line at three months indicates the 90-day acute/late toxicity boundary. Stage labels are shown where group staging was available or derivable from TNM components. n=10 patients with evaluable follow-up data. RT, radiation therapy; PBT, proton beam therapy; TNM, Tumor, Node, Metastasis

Durable local disease control was achieved, with no local or regional recurrences observed in any patient (100% local control rate). Distant metastases developed in one patient who subsequently died. Individual patient follow-up trajectories are illustrated in Figure 2. Among the nine curative-intent, non-metastatic patients with evaluable follow-up, two-year OS and PFS were both 100%, with no deaths or disease progression observed. Among the two patients with metastatic disease, one died during follow-up (at 28.3 months) following distant disease progression. The other metastatic patient had no follow-up data available.

Toxicity and safety profile

**Figure 3:**
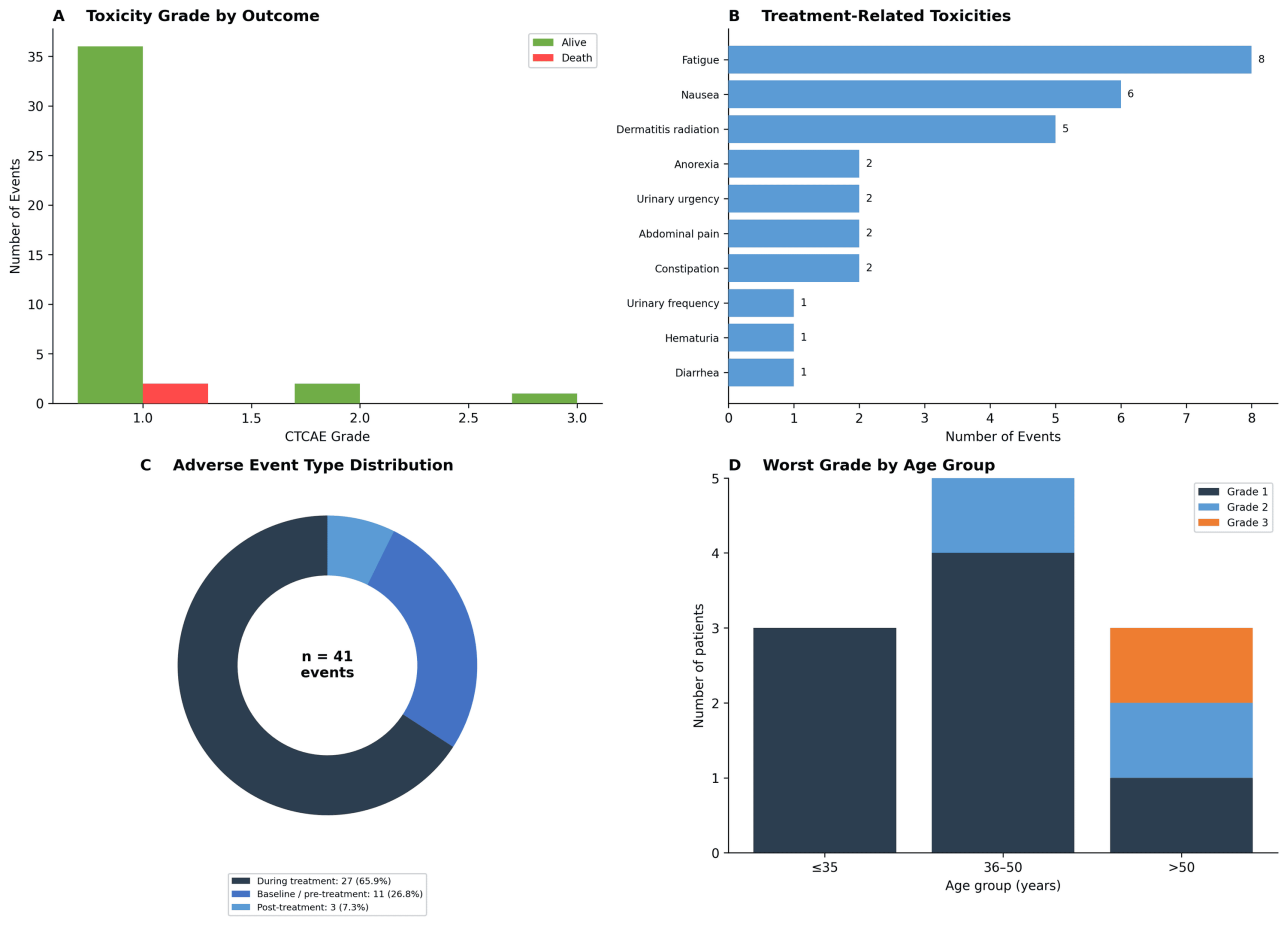
Adverse event characterization by patient outcome, age, and toxicity type (A) Distribution of CTCAE toxicity grades by patient vital status (alive vs. deceased). (B) Most common treatment-related toxicities ranked by number of events. (C) Donut chart showing the proportion of adverse events by timing/type (total=41 events). (D) Worst toxicity grade distribution stratified by age group. CTCAE, Common Terminology Criteria for Adverse Events

Adverse events are summarized in Table 4. The toxicity profile was favorable, with one grade 3 event occurring among the 41 total adverse events recorded. This grade 3 event was not treatment-related. No serious adverse events were reported. Most adverse events were grade 1, predominantly occurring in patients who remained alive (Figure 3A).

**Table 4:** Summary of adverse events Adverse events were graded according to CTCAE v5.0. The single grade 3 event (thromboembolic event) was not treatment-related. No serious adverse events were reported. CTCAE, Common Terminology Criteria for Adverse Events

Category	Grade 1	Grade 2	Grade 3	Total
Baseline/pre-treatment	11	0	0	11
During treatment	25	2	0	27
Post-treatment	2	0	1	3
Serious adverse event	0	0	0	0
Total	38 (92.7%)	2 (4.9%)	1 (2.4%)	41

Fifty-eight percent of patients (11/19, 58%) experienced at least one adverse event, with 11 (11/19, 58%) experiencing treatment-related toxicity. The remaining eight patients (8/19, 42%) had no documented adverse events. The most common treatment-related adverse events were fatigue, nausea, and radiation dermatitis, followed by abdominal pain, constipation, and urinary urgency (Figure 3B). Specifically, fatigue and nausea each occurred in six patients (6/19, 32%), whereas radiation dermatitis occurred in five (5/19, 26%). Additional treatment-related toxicities included anorexia and abdominal pain (2/19, 11% each). Gastrointestinal and genitourinary toxicities were generally mild and manageable, with 92.7% (38/41) of all adverse events being grade 1.

**Figure 4:**
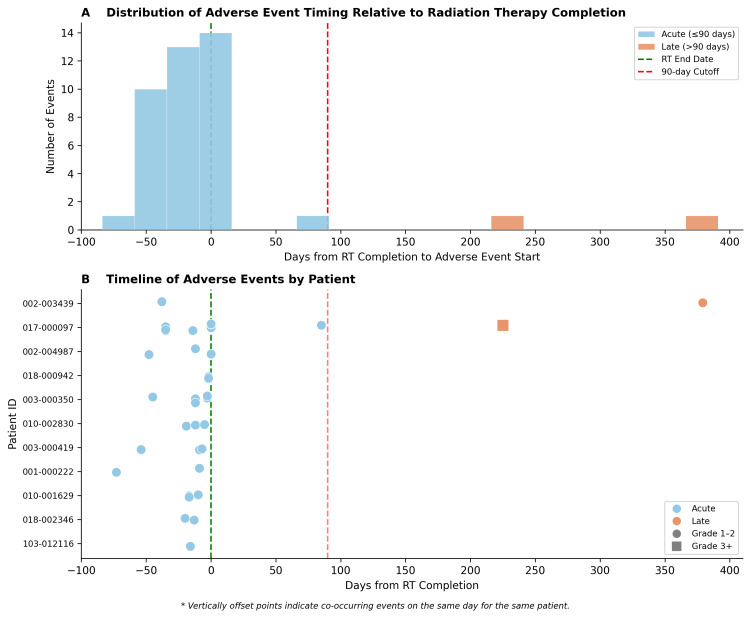
Adverse event timing relative to RT completion (A) Distribution of adverse event onset by days from RT completion. Blue = acute events (onset ≤90 days after RT end); orange = late events (onset >90 days after RT end). Green dashed line = RT end date (day 0); red dashed line = 90-day acute/late cutoff. (B) Timeline of individual adverse events by patient. Circles = grade 1-2 events; squares = grade 3+ events. Vertically offset points indicate co-occurring events on the same day for the same patient. n=11 patients with documented adverse event data; 41 total events. RT, radiation therapy

One grade 3 event (thromboembolic event) occurred in one patient (1/19, 5%) and was not related to RT. To distinguish acute from late toxicities, all adverse events were anchored to each patient's individual RT end date (Figure 4). Of 30 evaluable post-enrollment adverse events, 28 (28/30, 93%) were classified as acute (Figure 3C). All 28 acute events were grade 1-2, consisting predominantly of fatigue, nausea, and radiation dermatitis, and all resolved without treatment discontinuation or hospitalization. Eleven events recorded prior to RT initiation were classified as pre-existing baseline conditions and were not attributable to treatment.

Late toxicities were observed in two of 19 patients (2/19, 11%), comprising two events in total. In two patients with non-metastatic disease, late events were limited to a grade 1 urinary urgency (onset day 379 after RT end, no intervention required) and a grade 3 thromboembolic event (onset day 225). Toxicity grade distribution by age group is presented in Figure 3D.

Overall treatment tolerability was excellent, with predominantly grade 1-2 toxicities during active treatment and no treatment-related hospitalizations or treatment discontinuations due to toxicity. Functional status was well preserved, with 18 patients (18/19, 95%) maintaining ECOG performance status 0-1 at follow-up.

Secondary malignancy risk

No secondary malignancies were observed during the follow-up period, corresponding to zero events over 28.8 person-years of observation. However, this follow-up duration is insufficient to draw meaningful conclusions about secondary malignancy risk, as radiation-induced cancers typically emerge five to 20 years after treatment [6]. These early data serve as a baseline for ongoing surveillance of this prospective cohort, and future reports with longer follow-up will be essential to assess whether the dosimetric advantages of proton therapy translate into clinically meaningful reductions in secondary cancer risk. This compares favorably to predicted secondary cancer rates of two to eight excess cases per 100 person-years reported for photon-based radiotherapy in young seminoma patients [5].

## Discussion

This multi-institutional experience suggests that PBT for testicular seminoma achieves excellent clinical outcomes with a favorable toxicity profile. The data align with the theoretical advantages of proton therapy in this young patient population, where reducing long-term sequelae is a central concern.

The clinical outcomes in our series compare well with published data for both proton- and photon-based approaches. The 100% two-year OS and PFS among non-metastatic patients fall within the range of historical photon series reporting two-year survival rates of 85-95% [2], and the 100% local control rate is consistent with the known radiosensitivity of seminomatous tumors.

Recent comparative studies have found similar disease control with proton and photon approaches, indicating that the dosimetric benefits of proton therapy do not come at the cost of tumor control [8]. Our data reinforce this finding, with excellent disease control across the range of clinical stages in our cohort.

Key strengths of the present study include its prospective, multi-institutional registry design, which provides real-world outcomes data not subject to the selection and recall biases inherent in retrospective chart reviews. Additionally, our systematic adverse event characterization using CTCAE v5.0 with temporal classification anchored to individual RT end dates (Figure 4) provides a level of toxicity detail not previously reported for proton therapy in seminoma. Compared with Maxwell et al. (n=24, University of Pennsylvania, single-institution retrospective) [10] and Pasalic et al. (n=11 proton patients, MD Anderson Cancer Center, single institution) [8], our prospective data collection ensures standardized documentation. Prior reports either lacked prospective data collection or were limited to dosimetric comparisons or case reports without clinical outcomes (Efstathiou et al. [7], Hoppe et al. [9], Haque et al. [11], Rønde et al. [12]).

No grade ≥3 treatment-related adverse events occurred in our series, which compares well with historical photon therapy results, where grade ≥3 acute toxicity rates of 10-25% have been reported, mostly gastrointestinal and genitourinary in nature [13].

The predominantly grade 1-2 toxicity profile in our patients likely reflects the superior dose conformality of proton therapy. Dosimetric studies have consistently shown 40-60% reductions in mean doses to organs at risk (including the small bowel, bladder, and kidneys) when comparing proton with photon plans [12], and these dosimetric gains appear to translate into tangible clinical tolerability. We also did not observe any late radiation-specific complications such as bowel obstruction, ureteral stricture, or myelopathy, although longer follow-up will be needed to fully characterize the late toxicity profile. While proton therapy involves higher upfront costs compared with photon approaches, cost-effectiveness analyses have suggested that the reduced risk of secondary malignancies and late complications may result in long-term cost savings [14]. Given that seminoma patients are typically young and have decades of life ahead, even modest reductions in late morbidity could be clinically meaningful over a lifetime of survivorship.

The inclusion of two palliative-intent patients reflects real-world proton therapy practice. The dosimetric advantages of proton therapy, particularly reduced dose to organs at risk, may benefit palliative patients who have received or will receive systemic therapy, potentially minimizing cumulative organ toxicity. The small number of palliative patients precludes conclusions regarding relative benefit, and these patients are reported descriptively in Table 3.

The absence of secondary malignancies during our follow-up period is worth highlighting, even though the median follow-up of 24 months is still short for detecting second cancers. Population-based data suggest that excess secondary cancer risk begins to emerge within the first five years after radiotherapy [6].

Mathematical modeling studies by Simone and colleagues have estimated substantial reductions in secondary cancer risk with proton therapy, on the order of two to eight fewer excess secondary malignancies per 100 patients treated relative to photon approaches [5]. These predictions are derived from dose-response relationships established in epidemiological data, and our early clinical experience, while limited, is compatible with these projections.

The evolution from uniform scanning to PBS during our study period reflects improvements in proton therapy technology. PBS techniques are associated with superior dose conformality and reduced integral dose, potentially further improving the therapeutic ratio. The fact that all patients in our series completed treatment without interruption speaks to the reliability and practicality of modern proton therapy delivery.

Several limitations should be acknowledged. The PCG registry spans 14 years and multiple institutions, resulting in variability in data completeness, particularly for formal AJCC staging fields, which were less consistently populated during earlier enrollment periods and at certain contributing sites. While metastatic status was available for all patients, formal group staging remained unavailable for 32% of the cohort despite our best efforts to derive it from available TNM components. With only 19 patients and a relatively short median follow-up, we are underpowered to detect rare late effects and secondary malignancies. Additionally, one patient in our cohort had a non-seminomatous germ cell tumor (NSGCT) rather than pure seminoma histology. This was a palliative-intent patient retained to reflect real-world registry practice; all 17 curative-intent patients had confirmed seminoma. The heterogeneous patient population in terms of clinical stage (I-IIIC), treatment intent (curative and palliative), and metastatic status reflects real-world practice but complicates outcome interpretation and limits comparisons with more homogeneous published series. To mitigate this, survival analyses were restricted to the curative-intent non-metastatic cohort (n=17 treated, n=9 with evaluable follow-up), and palliative-intent and metastatic patients were reported descriptively and individually in Table 3. Given the rarity of proton therapy for seminoma, we believe that reporting the full cohort with appropriate stratification provides the most transparent and informative presentation of early clinical experience.

Our 19 patients were drawn from eight PCG member centers. Neither the University of Pennsylvania (Maxwell et al.) nor the MD Anderson Cancer Center (Pasalic et al.) is a PCG member site, making patient overlap with these prior cohorts unlikely. The only prior publication from a PCG-affiliated institution is the single case report by Haque et al. from ProCure Proton Therapy Center, which contributed patients to our cohort; overlap of at most one patient is possible but cannot be confirmed because of the de-identification of registry data.

The absence of a contemporary photon-treated control group limits direct comparative analysis. That said, our results can still be placed alongside historical photon therapy outcomes and contemporary published data. We also did not perform individual patient-level dosimetric comparisons between proton and photon plans, which would have further informed treatment selection. Finally, the short median follow-up relative to the latency period for radiation-induced secondary malignancies (typically five to 20+ years) limits our ability to draw conclusions about long-term secondary cancer risk, which represents one of the primary hypothesized advantages of proton over photon therapy in this population.

Continued follow-up of this cohort will be critical for understanding the late-effects profile and any reduction in secondary malignancy risk. Prospective randomized trials comparing proton and photon approaches would provide the highest level of evidence, although such studies face practical challenges given the relative rarity of seminoma and potential equipoise concerns in light of the dosimetric advantages of proton therapy in this predominantly young patient population.

Integration of modern photon techniques such as intensity-modulated radiotherapy (IMRT) and volumetric modulated arc therapy (VMAT) into comparative analyses will be important, as these approaches offer improved dose conformality over historical 3D conformal techniques. However, dosimetric comparisons continue to show advantages for proton therapy even against these modern photon techniques [15].

## Conclusions

In this prospective multi-institutional registry cohort, PBT for testicular seminoma achieved excellent disease control with minimal acute toxicity. The 100% local control rate, absence of treatment-related grade ≥3 adverse events, and favorable treatment tolerability support proton therapy as a feasible and well-tolerated option for seminoma. However, the small sample size and short median follow-up preclude conclusions regarding long-term safety or reductions in secondary malignancy risk. Continued surveillance of this cohort is essential to determine whether the dosimetric advantages of proton therapy translate into meaningful reductions in late morbidity for this young, highly curable patient population.

## References

[1] Shiao JC, Shen X (2024). Contemporary role of radiation therapy in testicular cancer. Urol Clin North Am.

[2] Garcia-Serra AM, Zlotecki RA, Morris CG, Amdur RJ (2005). Long-term results of radiotherapy for early-stage testicular seminoma. Am J Clin Oncol.

[3] Giannatempo P, Greco T, Mariani L (2015). Radiotherapy or chemotherapy for clinical stage IIA and IIB seminoma: a systematic review and meta-analysis of patient outcomes. Ann Oncol.

[4] Haugnes HS, Bosl GJ, Boer H (2012). Long-term and late effects of germ cell testicular cancer treatment and implications for follow-up. J Clin Oncol.

[5] Simone CB 2nd, Kramer K, O'Meara WP, Bekelman JE, Belard A, McDonough J, O'Connell J (2012). Predicted rates of secondary malignancies from proton versus photon radiation therapy for stage I seminoma. Int J Radiat Oncol Biol Phys.

[6] Travis LB, Fosså SD, Schonfeld SJ (2005). Second cancers among 40,576 testicular cancer patients: focus on long-term survivors. J Natl Cancer Inst.

[7] Efstathiou JA, Paly JJ, Lu HM (2012). Adjuvant radiation therapy for early stage seminoma: proton versus photon planning comparison and modeling of second cancer risk. Radiother Oncol.

[8] Pasalic D, Prajapati S, Ludmir EB, Tang C, Choi S, Kudchadker R, Frank SJ (2020). Outcomes and toxicities of proton and photon radiation therapy for testicular seminoma. Int J Part Ther.

[9] Hoppe BS, Mamalui-Hunter M, Mendenhall NP, Li Z, Indelicato DJ (2013). Improving the therapeutic ratio by using proton therapy in patients with stage I or II seminoma. Am J Clin Oncol.

[10] Maxwell R, Chang Y, Paul C, Vaughn DJ, Christodouleas JP (2023). Cancer control, toxicity, and secondary malignancy risks of proton radiation therapy for stage I-IIB testicular seminoma. Adv Radiat Oncol.

[11] Haque W, Wages C, Zhu XR (2015). Proton therapy for seminoma: case report describing the technique, efficacy, and advantages of proton-based therapy for seminoma. Pract Radiat Oncol.

[12] Rønde HS, Kronborg C, Høyer M (2023). Dose comparison of robustly optimized intensity modulated proton therapy (IMPT) vs IMRT and VMAT photon plans for testicular seminoma. Acta Oncol.

[13] Rosen DB, Tan AJN, Pursley J, Kamran SC (2023). Advances in radiation therapy for testicular seminoma. World J Urol.

[14] Verma V, Shah C, Rwigema JC, Solberg T, Zhu X, Simone CB 2nd (2016). Cost-comparativeness of proton versus photon therapy. Chin Clin Oncol.

[15] Pursley J, Remillard K, Depauw N (2024). Radiation therapy for stage IIA/B seminoma: modeling secondary cancer risk for protons and VMAT versus 3D photons. Cancers (Basel).

